# Single-Molecule Detection of Nucleic Acids via Liposome Signal Amplification in Mass Spectrometry

**DOI:** 10.3390/s22041346

**Published:** 2022-02-10

**Authors:** Xiangcheng Lin, Mengmeng Zhao, Mingyue Li, Juan Long, Jing Zhang, Fang Yu, Fen Xu, Lixian Sun

**Affiliations:** Guangxi Key Laboratory of Information Materials, School of Materials Science and Engineering, Guilin University of Electronic Technology, Guilin 541004, China; 15164661310@163.com (M.Z.); handsome_xiaoli@163.com (M.L.); ECKJ123456@163.com (J.L.); ZJ_1997_ZJ@163.com (J.Z.); yufang4599@163.com (F.Y.); xufen@guet.edu.cn (F.X.)

**Keywords:** mass spectrum, nucleic acid, signal amplification, liposome

## Abstract

A single-molecule detection method was developed for nucleic acids based on mass spectrometry counting single liposome particles. Before the appearance of symptoms, a negligible amount of nucleic acids and biomarkers for the clinical diagnosis of the disease were already present. However, it is difficult to detect extremely low concentrations of nucleic acids using the current methods. Hence, the establishment of an ultra-sensitive nucleic acid detection technique is urgently needed. Herein, magnetic beads were used to capture target nucleic acids, and liposome particles were employed as mass tags for single-particle measurements. Liposomes were released from magnetic beads via photocatalytic cleavage. Hence, one DNA molecule corresponded to one liposome particle, which could be counted using mass spectrometric measurement. The ultrasensitive detection of DNA (10^–18^ M) was achieved using this method.

## 1. Introduction

As one of the basic life molecules, nucleic acids play a pivotal role in the storage, transmission, and expression of genetic information. Nucleic acids are closely related to various clinical diseases and have become an important basis for their clinical diagnosis and treatment [1,2]. Currently, nucleic acid detection is performed via signal amplification methods. The polymerase chain reaction (PCR) is one of the most commonly used methods in clinical practice, which can amplify a target sequence more than a million times in a brief period and requires small sample volumes [3]. Other available techniques include the rolling circle amplification reaction (RCA) [4,5], the hybridisation chain reaction (HCR) [6], and loop-mediated isothermal amplification (LAMP) [7,8,9]. However, these techniques present a series of limitations. The sensitivity of PCR is hundreds of copies per ml, but PCR is not suitable for the detection of bioactive molecules because they involve a high-temperature denaturation process (~95 °C). The sensitivity of RCA is close to that of PCR, and the equilibrium time of RCA is about 6 h. In RCA, the need to synthesise cyclic single-chain DNA as a template increases the complexity of the experimental operation, thus limiting its practical application. HCR is a relatively simple amplification method under enzyme-free conditions, but the sensitivity is thousands of copies per ml. HCR cannot be used for the analysis of extremely low levels of nucleic acids. The equilibrium time of LAMP is about 1 h, and the sensitivity of LAMP reaches the level of PCR. The major problem with LAMP is that it cannot perform multiplex amplification.

Viruses are composed of nucleic acids (DNA or RNA) and proteins [10,11]. A variety of viruses are present in nature, some of which can cause serious diseases and damage to human health, for example, avian influenza virus (AIV), human immunodeficiency virus (HIV), and hepatitis B and C viruses (HBV, HCV) [12]. These diseases are usually highly contagious, with no effective cure. Therefore, the detection of viral nucleic acids is of the utmost importance in disease control. In the absence of symptoms, the number of copies of the infecting virus is still exceedingly small, and the detection of its nucleic acids is an extremely challenging task [13]. If the amount of viral nucleic acid could be detected at this time, a prompt and effective treatment can increase the chances for patient recovery. Therefore, there is an urgent need for ultra-sensitive detection methods.

The low ionisation efficiency and poor mass spectrometry detection sensitivity of single and double chains of nucleic acids have greatly limited their application [14,15]. In a recent study, DNA detection probes were labelled with rare earth elements; these were hybridised with target DNA chains on magnetic beads, with each element corresponding to different target DNA chains [16]. Direct detection with inductively coupled plasma mass spectrometry (ICP-MS) after cleaning the beads enabled the high-throughput analysis of multiple target DNA chains. The detection limit of target DNA chains reached 0.5 pM. Although this method improved the sensitivity of mass spectrometry for nucleic acids by several orders of magnitude, it still cannot be applied to the detection of lower levels of nucleic acid markers in clinical settings [17].

The atomisation efficiency of ICP-MS is limited; typically, it is only 0.5%, with the ICP ionisation process also resulting in the loss of many ions [18]. However, the flow path of electrospray ionisation mass spectrometry (ESI-MS) is simple [19]. The sample is discharged through a spray needle, and high-voltage and auxiliary gas are applied before the sample is atomised, ionised, and injected into the mass analyser for spectrometry. The high atomisation efficiency of ESI and the high ratio of ions entering the mass spectrum make ESI-MS have a higher detection rate.

Lin used liposomes to amplify the signal of protein biomarkers and detected them via surface-enhanced laser desorption ionisation mass spectrometry (SELDI-MS) [20]. The detection limit of proteins was only 0.2 ng/mL. Therefore, SELDI-MS cannot be applied to the detection of low-concentration nucleic acids. Phosphatidylcholine is highly ionised in ESI and has high sensitivity in the positive ion acquisition mode. The polar head of these phospholipids is a quaternary amine group, which is positively charged. Such phospholipids have been used as the main component to prepare liposomes, and the obtained liposomes produced strong signals in ESI-MS [21,22]. Based on the above assumptions, we labelled liposomes on the detection probe to amplify the signal of the target nucleic acids. Liposomes were counted via mass spectrometry to determine the number of target nucleic acids.

## 2. Materials and Methods

### 2.1. Reagents and Materials

#### 2.1.1. Materials

Distearoylphosphatidylcholine (DSPC), cholesterol, and polyethylene glycol-azide-modified distearoyl phosphatidylethanolamine (DSPE-PEG2000-N3) were purchased from Avanti. Gold nanoparticles (AuNPs, particle sizes of 20, 40, 60, and 80 nm) were purchased from BBI. All nucleic acid chains were synthesised by the Dalian Bao Biological Company (Table 1); streptavidin magnetic beads were purchased from Thermo Fisher, and chloroform was purchased from Shanghai Sinopharm. All the water used was ultra-pure water, with resistivity of ≥18.3 MΩ·cm.

#### 2.1.2. Instrumentation

A rotary evaporator (RE-2000A, Shanghai Lihua Instrument Equipment Co., Ltd., Shanghai, China), high-speed centrifuge (3K30, Beckman, CA, USA), field emission transmission electron microscope (Tecnai G2 F20, FEI, Hillsboro, OR, USA), nanoparticle size and zeta potential tester (ZS90, Malver, Worcestershire, UK), LTQ-Orbitrap MS (Velos Pro, Thermo Fisher, Waltham, MA, USA), ICP-MS (DRC-e, PE, USA), and probe ultrasound system (JY92-Π D, Ningbo Xinzhi Biological Technology Co., Ltd., Ningbo, China) were used.

### 2.2. Method

#### 2.2.1. Liposome Preparation

For liposome preparation, 3.12 mg of DSPC, 0.56 mg of DSPE-PEG2000-N3, 0.48 mg of cholesterol (with a molar ratio of 100:5:30, respectively), and 4 mL of chloroform were added to a round-bottom flask, mixed, and rotary evaporated under reduced pressure at 35 °C. A phospholipid film was formed on the inner wall of the flask that was purged in argon for 1 h to remove any residual solvent. Subsequently, 3 mL of AuNPs solution (with a particle concentration of 0.16 nM) was added to the round-bottom flask and shaken. The resulting liposomes were swelled in a water bath at 55 °C for 1 h. To ensure that a single gold nanoparticle was encapsulated in the liposomes, these were placed in an ice bath with an ultrasound probe (1 mm ultrasound probe, ultrasound power 100 W. Ultrasound 2 s, rest 5 s, with a duration of 1 h). Then, the liposomes were centrifuged at 4 °C for 30 min to remove the hollow liposomes in the solution’s upper layer. After several centrifugations, the liposomes were resuspended in 500 μL of phosphate-buffered saline (PBS). The obtained liposome–AuNPs complexes (hereinafter referred to as liposomes) were placed in a refrigerator at 4 °C for later use. During the preparation process, the loss of gold nanoparticles was negligible, and the final liposome concentration was 0.96 nM.

#### 2.2.2. Liposome-Labelled Detection Probe

The azide group of DSPE-PEG2000-N3 in liposome can react with the alkynyl group of the optical cleavage probe [23]. Briefly, 100 μL of copper sulphate solution (50 mM), 100 μL of bathophenanthroline disodium sulfonate solution (100 mM), 100 μL of sodium ascorbate solution (500 mM), and a 0.1 optical density (OD) optical cleavage probe (0.31 nmol) were added to 500 μL of liposome solution, magnetically stirred, and protected from light for 1 h. After the reaction, the mixture was centrifuged at 4 °C for 30 min to remove excess reagents. The centrifugation was repeated several times to resuspend the labelled liposomes in 500 μL of PBS.

**Figure 1:**
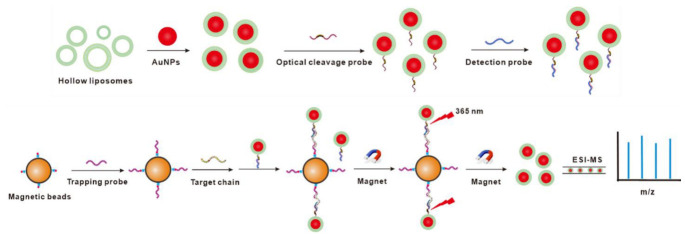
Schematic diagram of nucleic acid detection via liposome signal amplification in mass spectrometry.

After labelling the liposome with the optical cleavage probe, this was hybridised with a detection probe (Figure 1). One end of the detection probe was complementary to an optical cleavage probe, and the other end was complementary to the target chain. A total of 30 μL of detection probe (100 μM) was added to the 500 μL of labelled liposome solution and incubated for 2 h at 25 °C. At the end of the reaction, the liposomes were centrifuged and resuspended in 500 μL of PBS to obtain liposomes labelled with the detection probe.

To investigate the concentration of the labelled detection probe, the detection probe was replaced with fluorescence isothiocyanate (FITC) probe and hybridised with the optical cleavage probe on the liposome. The FITC concentration was then measured to determine the number of labelled liposomes. The procedure was as follows: 50 μL of photolysis probe-labelled liposomes (60 nm particle size) was mixed with 3 μL of FITC (100 μM) and incubated at 25 °C for 2 h. After the reaction, the unhybridised FITC probe was removed with several centrifugations. Then, 100 μL of potassium cyanide solution (10 mM) and 100 μL of sodium bicarbonate solution (5 mM) were added to the precipitate obtained via centrifugation. The gold nanoparticles were dissolved, and the fluorescence intensity of the solution was detected after the red colour of the gold nanoparticles was completely removed. A standard curve was drawn with different concentrations of FITC probe solutions to determine the concentration of probe bound to the liposomes. The results showed that the concentration of the probe was 190 nM in 500 μL of labelled liposomes.

#### 2.2.3. Magnetic Beads Labelled with Trapping Probe

Streptavidin magnetic beads (200 μL, 10 mg/mL) were cleaned three times with PBS. Then, 200 μL of trapping probe (10 μM was complementary to the target chain) was added and incubated at 25 °C for 1 h. The unbound trapping probe was removed via magnetic separation. The cells were washed with PBS 5 times and resuspended in 200 μL of PBS. According to the product specification, 1 mL of magnetic beads binds approximately 200 pmol of single-chained DNA. Therefore, in 200 μL of trapping probe and magnetic bead solution, the concentration of trapping probe was approximately 200 nM.

#### 2.2.4. Capture of Target Chain and Release of Liposome

The sample (20 μL) was placed in a centrifuge tube and incubated in a 65 °C water bath for 5 min, then quickly transferred to an ice bath. An amount of 5 μL of magnetic beads labelled with the trapping probe and 5 μL of liposomes labelled with the optical cleavage probe were added to the sample. The centrifuge tube was mixed well at 25 °C and reacted for 2 h. After magnetic separation, the sample was washed five times with PBS and resuspended in 80 μL of pure water. The obtained magnetic bead solution was irradiated with ultraviolet light for 10 min to cleave a photocleavable linker (PC linker) of the liposome surface (wavelength 365 nm, power 1.06 W). The magnetic beads were removed from the solution on the magnetic frame, and the remaining solution was used for mass spectrometry.

## 3. Results and Discussion

### 3.1. Principle of the Experiment

The ESI is a “soft” ionisation method that is suitable for the analysis of polar compounds and thermally unstable substances. Given the rapid development, the ESI can be combined with liquid phase separation equipment at various flow rates [24]. Phosphatidylcholine has a high sensitivity to ESI [25] and can be arranged into liposomes to amplify the signal of the measured substance. Liposomes are usually vesicles of varied sizes, with particle sizes ranging from tens to thousands of nanometres. To ensure the comparability of the mass spectrum signal, liposomes must be as similar as possible in size. The shape similarity of particle size can be improved with ultrasonic treatment, but the effect is not ideal. In our case, we achieved the control of liposome size through the size of the core AuNPs, greatly improving the degree of similarity in the shape of liposomes. Liposomes reacted with an optical cleavage probe containing PC linker via click chemistry. The other end of the optical cleavage probe was complementary to the detection probe, and the labelling was completed via chain hybridisation. Thus, the liposomes were tagged with both optical cleavage probes and detection probes. The other end of the detection probe was complementary to the target chain. To improve the photocleavage efficiency, two photocleavage sites were modified on the optical cleavage probe. The trapping probe on the magnetic bead was modified to be complementary to the target chain. Liposomes and magnetic beads were connected through the target chains. Magnetic separation removes excess liposomes, resulting in one target chain for each liposome. One target chain corresponds to one liposome. The number of mass spectrum peaks from liposomes is equal to the molecules of target chain. So, the amount of target chain was determined by measuring the number of liposomes via mass spectrometry (Figure 1). The higher the detection rate of liposome, the higher the detection sensitivity of the target chain.

### 3.2. Liposome Characterisation

**Figure 2:**
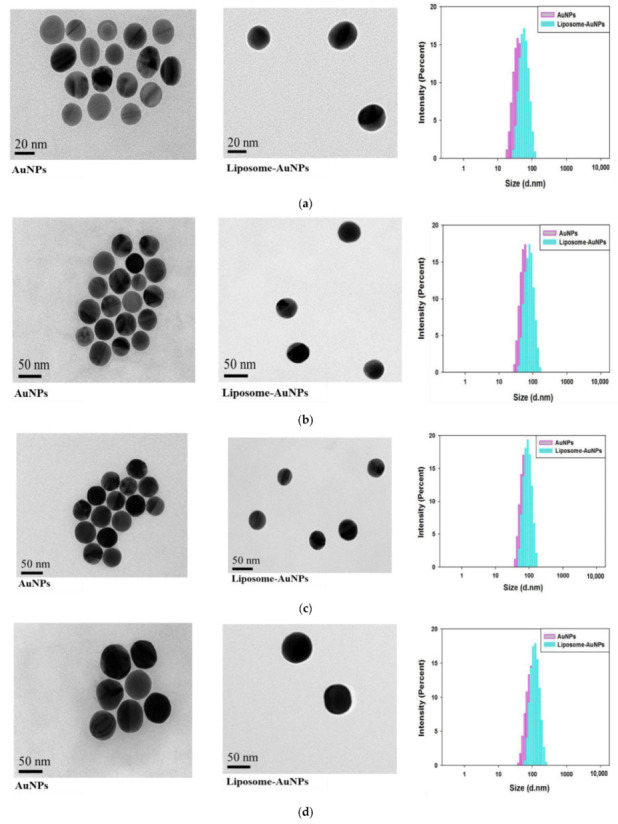
TEM data and DLS data of AuNPs with different sizes and their encapsulation by phospholipids: (**a**) 20 nm; (**b**) 40 nm; (**c**) 60 nm; (**d**) 80 nm.

Liposomes were prepared with the thin-film dispersion method. The phospholipids were first made into uniform thin film; then, the AuNPs solution was added. When the film encountered the aqueous solution, they peeled off from the inner wall of the round-bottomed flask and automatically formed lipid bilayer balls. During the formation of the lipid bilayer, AuNPs were encapsulated in a small ball, resulting in liposomes with a relatively similar size. The TEM images (Figure 2) suggest that, after AuNP wrapping, a 3–5 nm outer phospholipid layer was formed. However, in AuNPs that were not wrapped by phospholipids, no obvious outer layer was seen. The particle size distribution was determined via dynamic light scattering (DLS) (Figure 2). When AuNPs of different sizes (20, 40, 60, and 80 nm) were coated with phospholipids, the size of liposomes increased, with an overall increasing shift in size distribution. Hollow liposomes were distributed in the range of tens to thousands of nanometres. No particles of other sizes were observed in the DLS data, indicating that the hollow liposomes had been removed via centrifugation. Figure S1 shows the zeta potentials of AuNPs and liposomes. The potential of the AuNPs was negative 25 mV, while the zeta potential was negative 38 mV with phospholipidic coating. This indicates that the prepared liposomes have better dispersion and are suitable for stable preservation.

### 3.3. Selection of Liposome Particle Size

**Figure 3:**
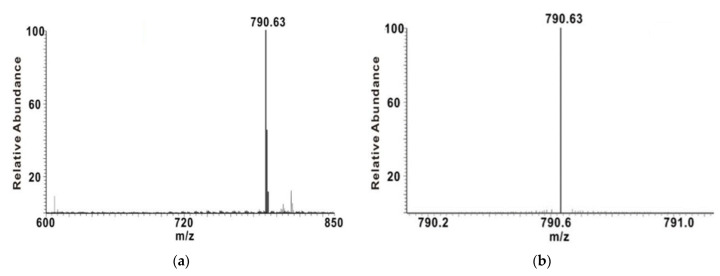
ESI mass spectra of DSPC: (**a**) detection in full-scan mode; (**b**) detection in selected ion-scan mode.

The mass spectrum signal of individual particles must be sufficiently strong to ensure an accurate count of individual particles. Four kinds of liposomes with different particle size were selected to detect the signal intensity of liposomes via mass spectrometry. The mass spectrometry detection was performed in the positive ion mode, and the ion peak with the strongest signal was DSPC [M+H]^+^ (790.63), which was selected to monitor the signal of the liposomes (Figure 3a). To eliminate the interference of unrelated ions and improve the signal-to-noise ratio, selected ion-scan mode was performed for mass spectrometry data collection (Figure 3b).

**Figure 4:**
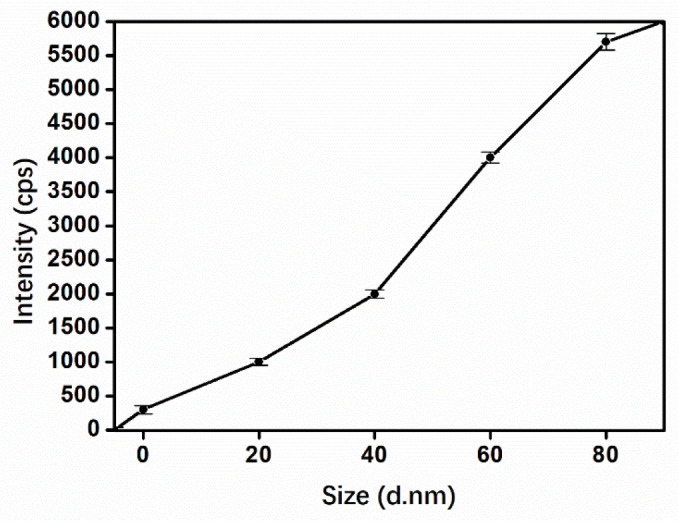
Plot of the intensity of the MS signal versus the size of the liposome.

Liposomes of different sizes were inserted directly into the spectrometer using an injection pump at a flow rate of 3 μL/min. When the flow rate was low, the sheath gas flow rate was reduced to avoid ion loss, and the auxiliary gas was turned off. Concomitantly, the distance between the injector and vacuum inlet of the mass spectrometer was adjusted. The sample was diluted to a certain concentration, and the liposomes were successively inserted into the mass spectrometer to obtain their respective signals, with each peak representing a particle. Pure water was used as a blank sample for testing, and the obtained background was approximately 400 cps (Figure S2a). As shown in Figure S2b, some signals of liposomes with 20 nm cores are only 1000 cps, but several are mixed with the background and cannot be distinguished. The signals of liposomes with 40 nm cores can reach 2000 cps, but some are still around 1000 cps, easily covered by the background noise. When liposomes with particle sizes of 60 nm were detected, most of the signal values were at 4000 cps, and the signal-to-noise ratio was approximately 10, which clearly was distinguished from the background; the number of signal peaks gradually increased with the increase in liposome particle size (Figure 4). Under this condition, the background noise can be ignored. When the particle size increased to 80 nm, the signal also improved. However, the dispersion of liposomes decreased slightly. After comprehensive consideration, 60 nm AuNPs were used as the particle of the liposomes.

Mass spectrum peaks with a signal-to-noise ratios higher than three times can be considered as the ionic peaks of DSPC. The ion peak heights are different in liposomes, which is because the ionisation efficiency of each particle is not the same.

### 3.4. The Effect of Polyethylene Glycol (PEG)

It has been reported that PEG polymers are hydrophilic and can reduce the adsorption of nucleic acids, proteins, and other substances on the nanoparticle surface [26,27,28]. The effect of DSPE-PEG2000-N3 on liposome dispersion was investigated by adding different proportions of DSPE-PEG2000-N3 as a liposome component.

When DSPE-PEG2000-N3 was not added, some of the liposomes agglomerated and formed uneven large particles, resulting in higher signal values (Figure S3a). After the addition of 2.5% DSPE-PEG2000-N3, the liposome dispersion greatly improved (Figure S3b), and no particularly strong ion peaks were detected. When 5% and 7.5% DSPE-PEG2000-N3 were added, the mass spectrum peak intensity of liposomes varied stably (Figure S3c,d). Optimal dispersion was achieved when 5% DSPE-PEG2000-N3 was added to the liposomes. If the DSPE-PEG2000-N3 content continued to increase, the hydrophilicity would also increase, which was not conducive to maintaining the stability of liposomes. Based on these experimental results, 5% DSPE-PEG2000-N3 was selected as the liposome component. The added DSPE-PEG2000-N3 not only regulated the dispersion of liposomes but also acted as the anchor point of the PC linker.

### 3.5. Assessment of the Number of Particles

It has been reported that AuNPs have high response values in ICP-MS and are easy to detect [29,30]. The AuNPs solution (5.0 × 10^5^ units/mL) was analysed via ICP-MS. In data, the ion peaks with counts of >10 were attributed to the AuNPs. A total of 4293 AuNPs signal peaks were detected with a 0.53% detection rate (Figure S4a). After wrapping the AuNPs with phospholipids, the hollow liposomes were removed via centrifugation to leave those with AuNPs at their cores. Liposomes with AuNP cores were prepared with the same concentration (5.0 × 10^5^ units/mL), and ICP-MS data were collected with the same parameters. In the data for liposomes (Figure S4b), there were 4133 mass spectrum peaks with counts of >10. The detection rate of liposomes was 0.51%.

The concentration of liposomes was 2.5 × 10^4^ units/mL when the liposome solution was diluted 20 times. The number of mass spectrum peaks of liposomes via ESI-MS was 746 (signal-to-noise ≥ 3), with a 99.5% detection rate (Figure S4c). These results indicate that the detection rate of liposomes via ESI-MS was much higher than that via ICP-MS.

In liposomes, the surface area of the polar heads of DSPC, DSPE-PEG2000-N3, and cholesterol, respectively, are 0.71, 0.41, and 0.19 nm^2^ [31,32,33]. In the prepared liposomes, the molar ratio of DSPC, DSPE-PEG2000-N3, and cholesterol was 100:5:30. Thus, the relative surface area of the polar head of the phospholipid molecule was 0.58 nm^2^. According to calculations, there are approximately 33,000 DSPC molecules in single liposome surfaces with 60 nm cores. A possible reason for the high detection rate of ESI-MS is that the liposomes are larger and contain more DSPC molecules. Even a fraction of DSPC ions entering the mass spectrometer can produce a signal strong enough to be three times higher than the background. Furthermore, the ESI nozzle is close to the vacuum inlet of the mass spectrometer; the ions generated by each liposome can enter the mass spectrometer with high efficiency.

### 3.6. ESI-MS Detection of Liposomes at Different Concentrations

A solution of liposomes with 3.75 × 10^4^ units/mL was prepared and diluted 2, 5, 25, and 125 times. The number of liposomes were identified via ESI-MS with flow injection, and the results are shown in Figure S5; the number of mass spectrometry signal peaks increased linearly with increasing liposome concentration. In the detection range, the number of liposome signals showed a good linear relationship with liposome concentration (Figure S6). The linear equation is: Y = 4.4111X − 0.9276, correlation coefficient R^2^ = 1. This indicates that the detection rate of liposomes can be maintained at a stable level for different concentrations.

### 3.7. Target DNA Chain Detection

To validate the experimental protocol, we synthesised a target chain (Table 1), the sequence of which is consistent with a fragment of an HCV conserved region [34,35]. The designed target chain, trapping probe, and detection probe were verified using the NCBI gene bank, and the results showed that the designed nucleic acid sequence is highly specific to HCV nucleic acids.

**Figure 5:**
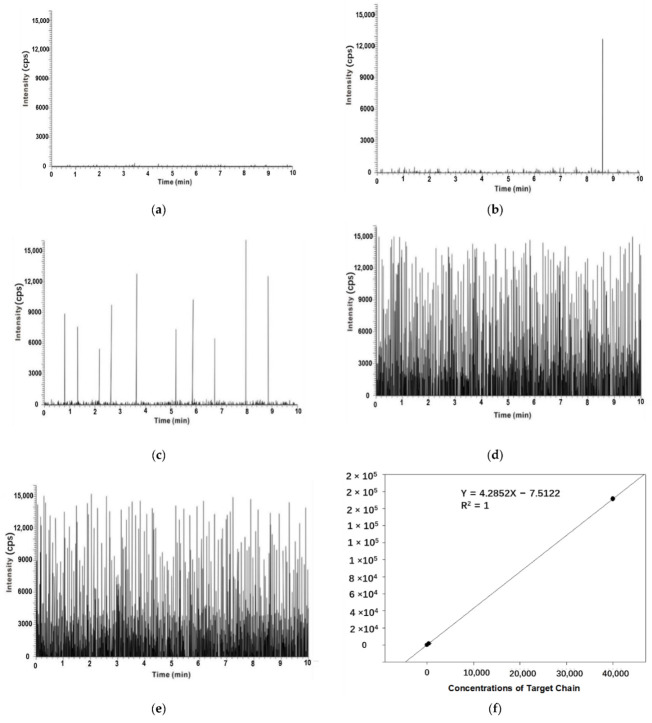
Mass spectrometry counting assay of target DNA: (**a**) pure water; (**b**) non-complementary DNA chain; (**c**) 4 aM DNA target chain; (**d**) 0.4 fM DNA target chain; (**e**) 40 fM DNA target chain; (**f**) plot of the number of liposomes versus the sample concentration.

The mass spectrometry detection of a pure water, non-complementary chain and different concentrations of target chain was examined. When pure water was used as a sample, no characteristic peaks of the liposomes were detected (Figure 5a). When detecting the non-complementary target chain, a single liposome signal peak was detected within 10 min (Figure 5b). When the target chain concentration was 4 aM, 10 liposome signal peaks were detected (Figure 5c). The theoretical number of peaks detected for the target chain of 4 aM DNA was 18, and the mass spectrometry detection rate was calculated to be 55.6%. When the sample concentration was increased to 0.40 fM, the number of liposomes detected was 845 × 2 = 1690 (Figure 5d). The theoretical value was 1800 liposomes, and the detection rate was 93.9%. When the sample concentration was increased to 40 fM, the number of liposomes detected was 857 × 200 = 171,400 (Figure 5e). The theoretical value was 180,000 liposomes, so the detection rate was 95.2%. The concentration of the target chain shows a good linear relationship with the number of peaks (Figure 5f). The linear equation is: Y = 4.2852X − 7.5122, correlation coefficient R^2^ = 1. With the increase in the target chain concentration, the detection rate improved. According to the experimental results, 10 out of 18 DNA molecules were detected. The minimum detection limit of the established mass spectrometry counting method was in the aM range, reaching the single-molecule level.

The zeta potential of the magnetic beads was negative 28 mV, while the zeta potential of the magnetic beads was negative 35 mV after trapping probe labelling (Figure S7). As shown in Figure S1, the zeta potential of liposomes was negative 38 mV, resulting in the automatic release of liposomes from the beads due to the negative zeta potential of both the liposomes and the magnetic beads.

## 4. Conclusions

Phosphatidylcholine phospholipids are easily ionised in ESI, with high sensitivity for mass spectrometry detection. The assembly of such phospholipids in liposomes as signal tags for nucleic acid detection enables mass spectrometric counting of target nucleic acids at the single-molecule level. In addition, different-quality labels can be obtained by using phospholipids of different molecular weights to prepare corresponding liposomes. So, liposomes with different components can tag different target chains, which can be used for the simultaneous detection of multi-component nucleic acids. Mass spectrometers have the characteristics of high accuracy, sensitivity, rapidity, and high throughput, and are now routine instruments in clinical medicine. The mass spectrometry method established in this study can be used for the detection of nucleic acid biomarkers at extremely low concentrations (such as circulating tumour nucleic acids), which will greatly aid in the prevention and treatment of diseases.

## Figures and Tables

**Table 1:** Synthetic oligonucleotide sequences.

Probes	Sequence (5′ to 3′)
Optical Cleavage Probe	alkynyl-TTTTTTTT-PC-TTTT-PC-TTTTTTGGCAGCACCGACGTAGAC
Detection Probe	CCAACACTACTCGGCTAGCAGGTCTACGTCGGTGCTGCC
Trapping Probe	Biotin- TTTTTTTTTTGTACCACAAGGCCTTCGCGA
Target Chain	CTGCTAGCCGAGTAGTGTTGGGTCGCGAAGGCCTTGTGGTAC
Non-Complementary Target Chain	CGCGATGTTTTGGCGCGTTAGTTCGTTGCGTATATTTTGTTG
FITC Probe	FITC-AAAAAGTCTACGTCGGTGCTGCC

## Data Availability

Not applicable.

## Supplementary Materials

The following are available online at https://www.mdpi.com/article/10.3390/s22041346/s1. Figure S1: zeta potentials of AuNPs and liposomes; Figure S2: mass spectra of AuNPs of different sizes after being wrapped by phospholipids: (a) blank, (b) 20 nm, (c) 40 nm, (d) 60 nm, and (e) 80 nm; Figure S3: mass spectra of liposomes with different PEG ratios: (a) 0%; (b) 2.5%; (c) 5%; (d) 7.5%; Figure S4: mass spectrometry assessment of particle numbers. (a) ICP-MS detection of AuNPs, (b) ICP-MS detection of AuNPs after being wrapped by phospholipids, (c) ESI-MS detection of AuNPs after being wrapped by phospholipids; Figure S5: ESI-MS assay of different concentrations of liposomes. (a) diluted × 125, (b) diluted × 25, (c) diluted × 5, (d) diluted × 2, (e) undiluted; Figure S6: linearity of the number of MS peaks versus concentration of liposomes; Figure S7: zeta potential of magnetic beads and magnetic beads labelled with trapping probe.
