# Supplementary material for: Single-Molecule Detection of Nucleic Acids via Liposome Signal Amplification in Mass Spectrometry
Source: Sensors (Basel). 2022 Feb 10;22(4):1346. doi: 10.3390/s22041346 (PMC8963037; doi:10.3390/s22041346)
Supplement: Supplementary file 1 [file sensors-22-01346-s001.zip › sensors-1538068-supplementary.pdf]

Supporting information

Results and discussion.

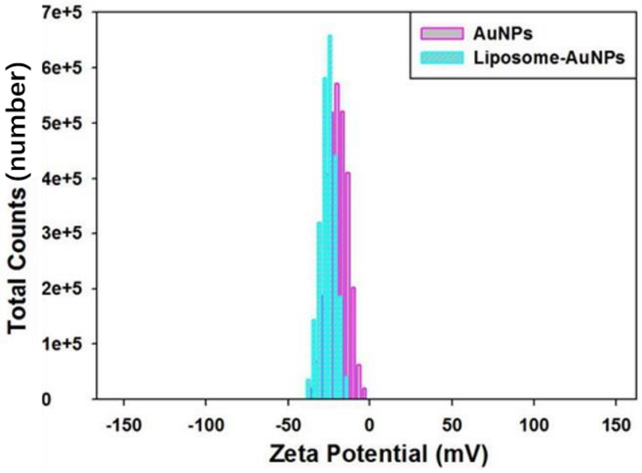

Figure S1. Zeta potentials of AuNPs and liposomes.

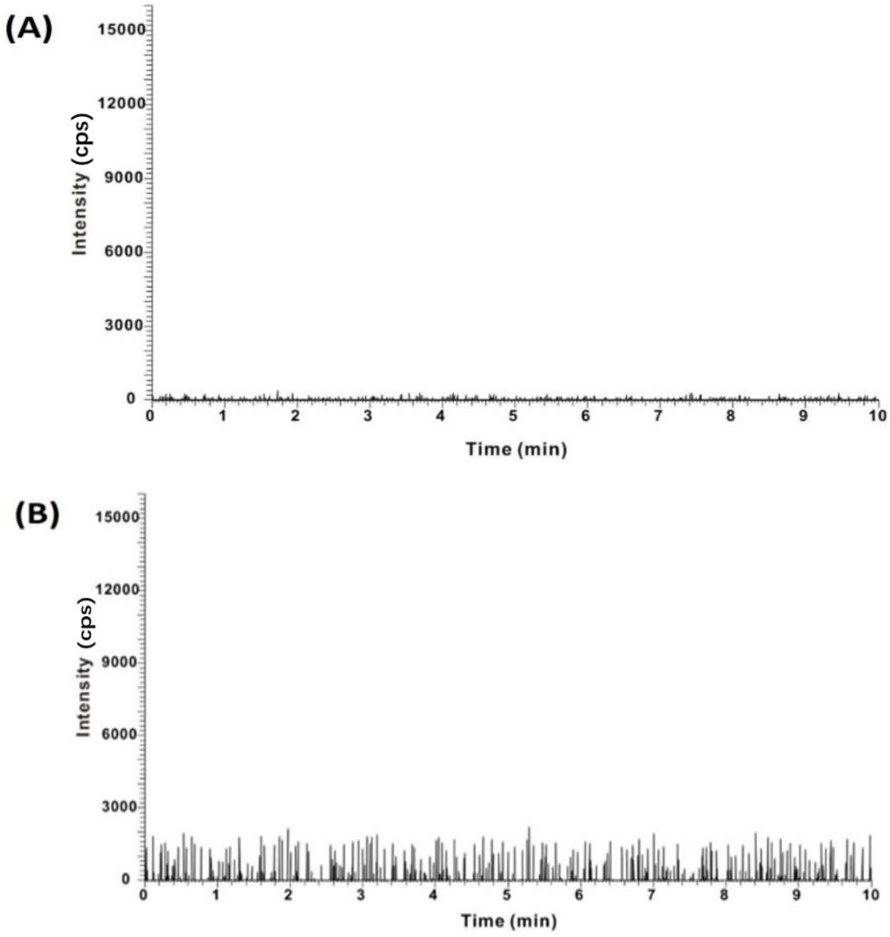

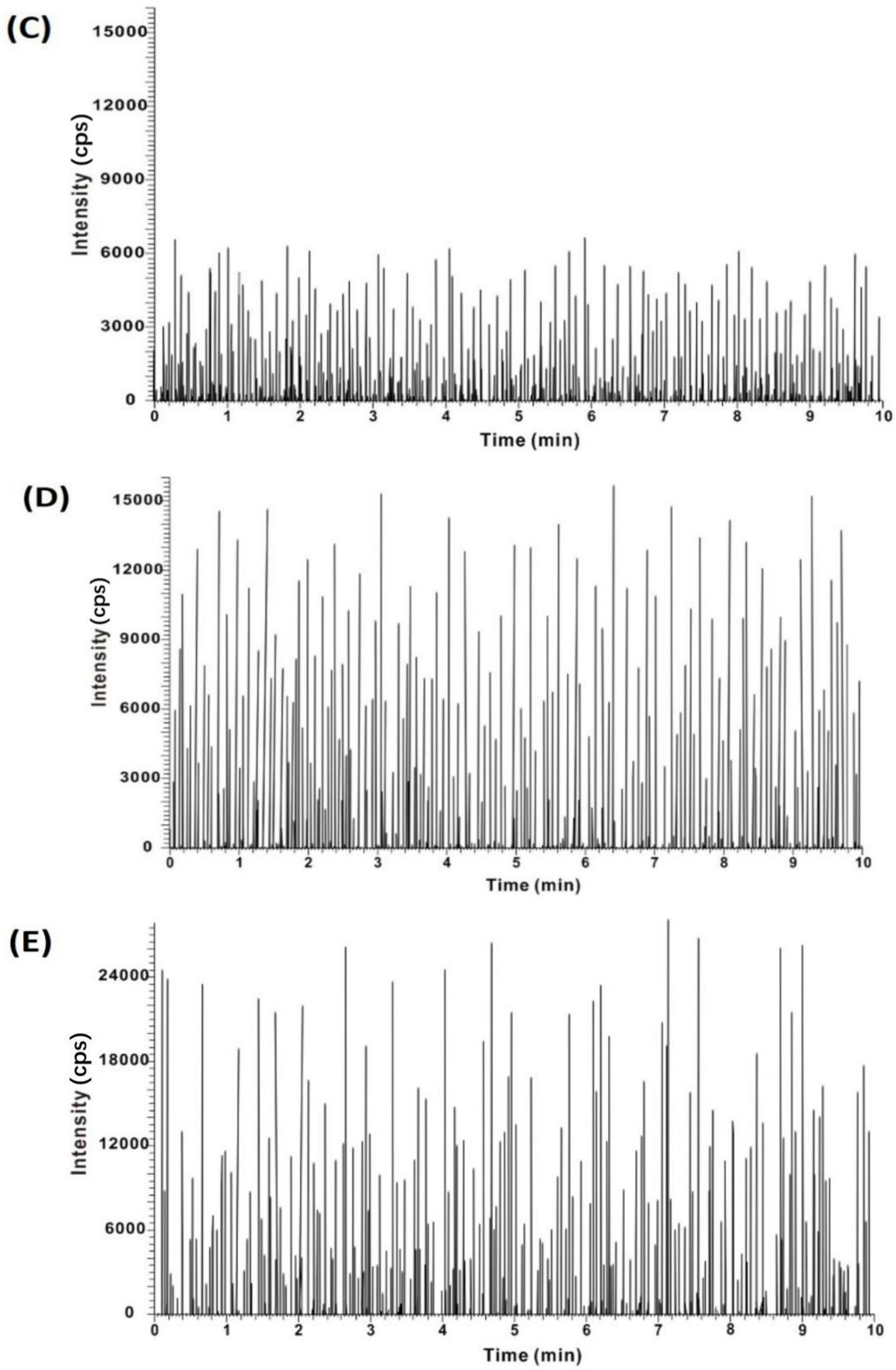

Figure S2. Mass spectra of AuNPs of different sizes after being wrapped by phospholipids: (a) blank; (b) 20nm; (c) 40nm; (d) 60nm; (e) 80nm.

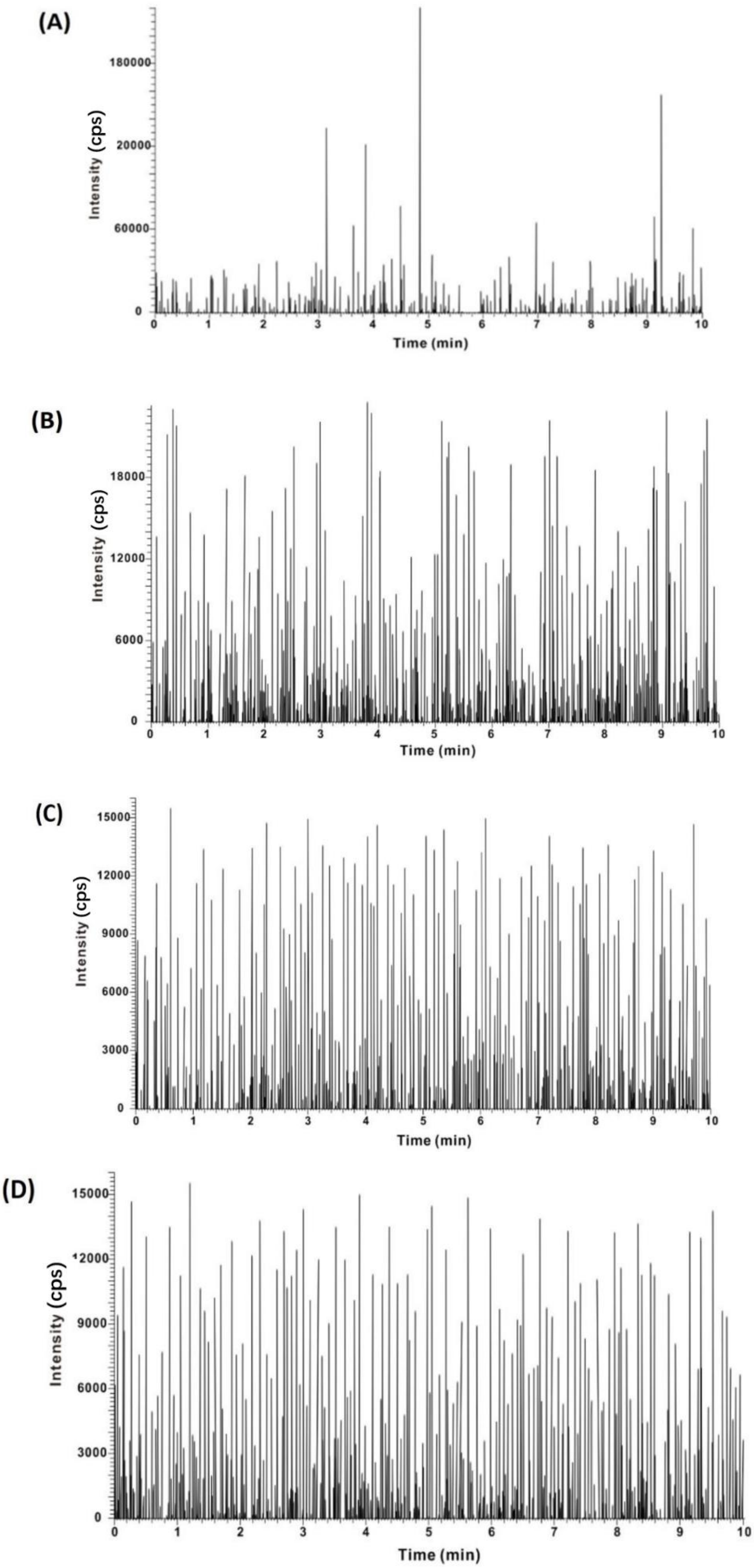

Figure S3. Mass spectral of liposomes with different PEG ratios: (a) 0%; (b) 2.5%; (c) 5%; (d) 7.5%.

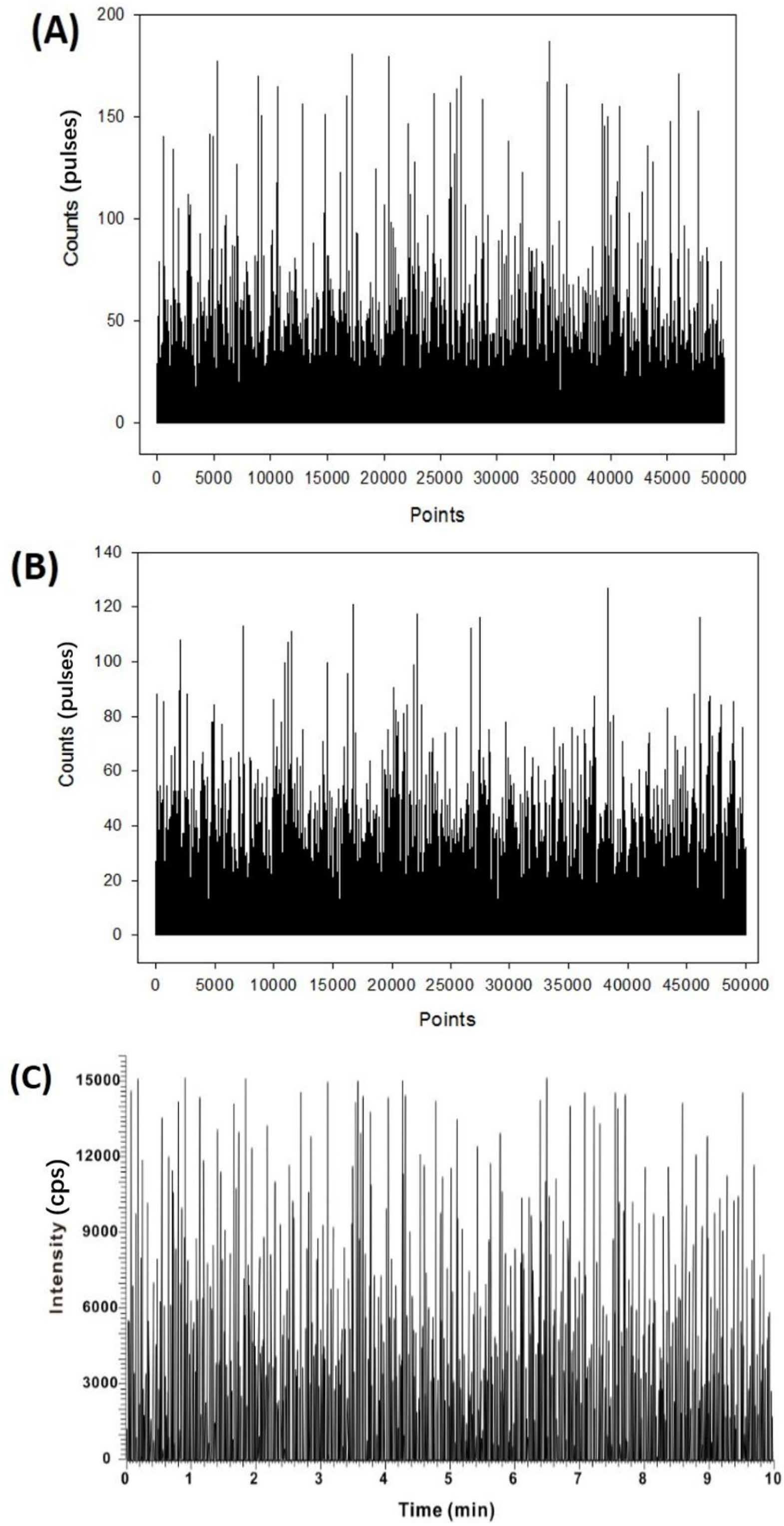

Figure S4. Mass spectrometry assessment of particle numbers. (A) ICP-MS detection of AuNPs, (B) ICP-MS detection of AuNPs after being wrapped by phospholipids, (C) ESI-MS detection of AuNPs after being wrapped by phospholipids.

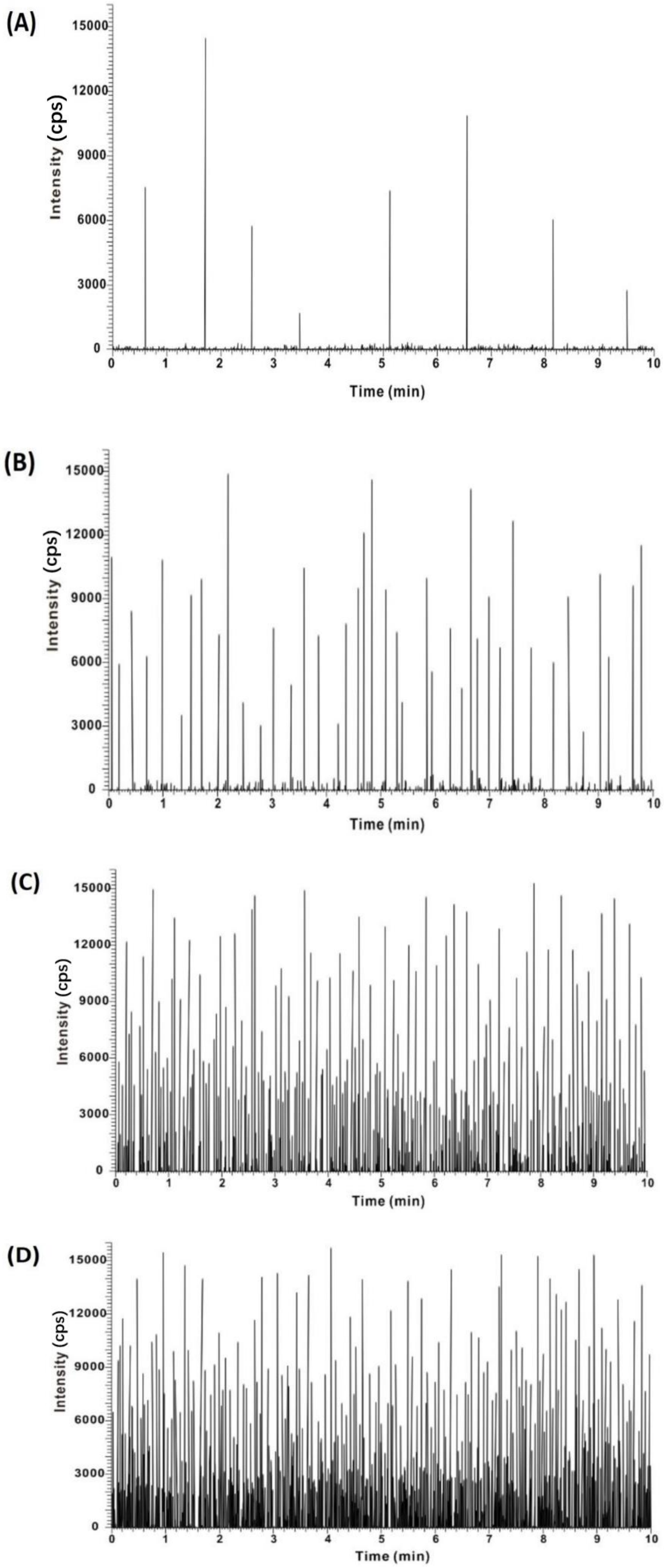
(A)
Intensity (cps)
Time (min)
(B)
Intensity (cps)
Time (min)
(C)
Intensity (cps)
Time (min)
(D)
Intensity (cps)
Time (min)

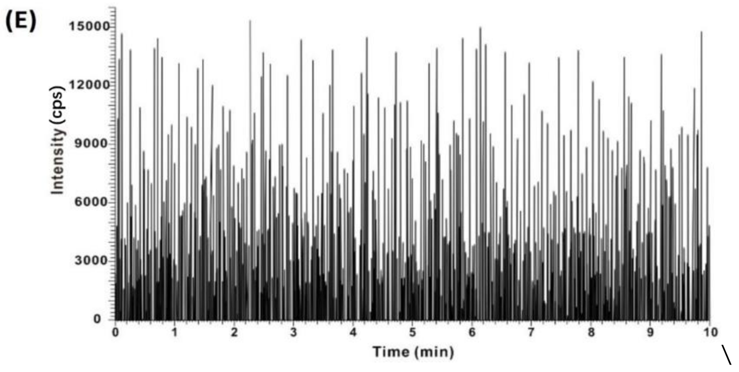

Figure S5. ESI-MS assay of different concentrations of liposomes. (A) diluted × 125, (B) diluted × 25, (C) diluted × 5, (D) diluted to × 2, (E) undiluted.

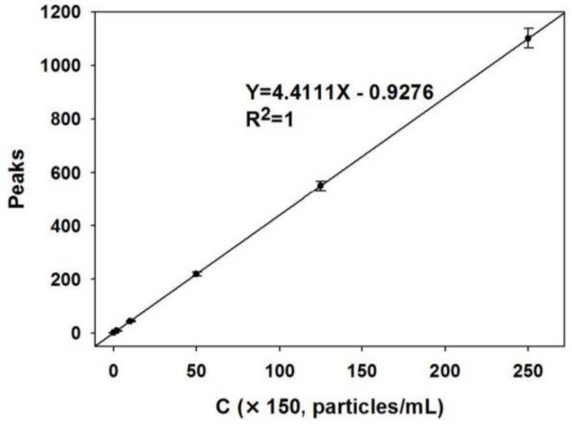

Figure S6. linearity of the number of MS peaks versus concentration of liposomes.

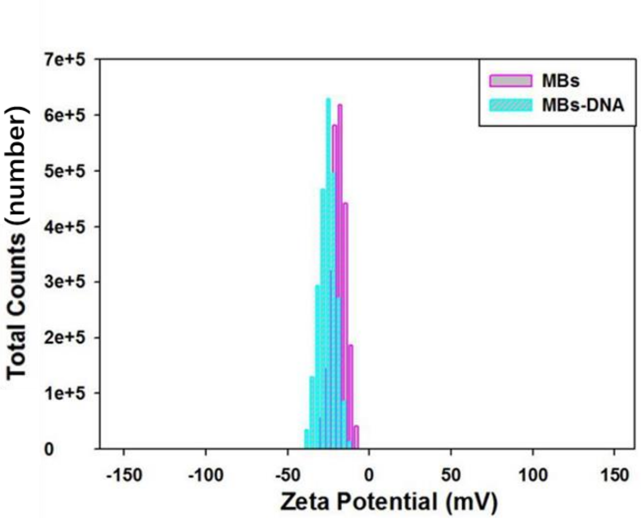

Figure S7. Zeta potential of magnetic beads and magnetic beads labelled with trapping probe.
